# Decompressive Craniectomy With Abdominal Bone Flap Preservation in Herpes Simplex Encephalitis: A Case Report

**DOI:** 10.7759/cureus.95262

**Published:** 2025-10-23

**Authors:** Elizabeth Blanco Espinosa, Idalberto Luis Fernandez Eng, Idania Maria Cruzata Matos, Javier Peña, Ricardo Marlon Saro Del Valle

**Affiliations:** 1 General Practice, CEDA Orthopedic Group, Miami, USA; 2 Surgery, Neurosurgery, Hospital Arnaldo Milian, Santa Clara, CUB; 3 Emergency, Hospital Universitario de la Ribera, Valencia, ESP; 4 General Medicine, HCA Healthcare, Nevada, USA; 5 Family Medicine, Universidad de la República (UDELAR), Montevideo, URY; 6 Neurology, Hospital General Dr. Juan Bruno Zayas Alfonso, Santiago de Cuba, CUB

**Keywords:** acyclovir therapy, autologous bone flap abdominal bone storage, cranial reconstruction, decompressive craniectomy, herpes simplex encephalitis (hse), intracranial hypertension, pediatric neurosurgery, refractory intracranial pressure, severe viral encephalitis, uncal herniation

## Abstract

Herpes simplex encephalitis (HSE) is a rare but severe condition that can lead to significant morbidity and mortality. Although antiviral therapy with acyclovir has improved outcomes, some patients develop complications such as focal hemorrhagic necrosis and edema in the temporal lobe, with subsequent elevation of intracranial pressure (ICP) and uncal herniation, necessitating surgical intervention. We report a pediatric case of HSE with life-threatening ICP refractory to sedation, osmotic therapy, and moderate hyperventilation. Despite aggressive treatment, the patient underwent a left-sided decompressive hemicraniectomy, with the bone flap preserved in a subcutaneous abdominal pocket, a technique shown in previous studies to maintain bone viability and minimize infection risk. This approach effectively controlled ICP and allowed progressive neurological recovery. At three-month follow-ups, the patient demonstrated favorable neurological outcomes (Glasgow Outcome Scale = 5) with successful autologous cranial reconstruction.

## Introduction

Herpes simplex encephalitis (HSE) is an acute infection of the central nervous system caused by the herpes simplex virus (HSV) types 1 or 2, characterized by inflammation and necrosis of the brain parenchyma [1]. It is the most common cause of sporadic, fatal viral encephalitis in individuals older than six months in developed countries, with an estimated annual incidence of two to four cases per million worldwide [2]. The disease affects both children and adults, with no clear seasonal or gender preference [3]. Without antiviral therapy, mortality approaches 70% and fewer than 3% of survivors retain normal neurological function [4]. The introduction of intravenous acyclovir has markedly improved outcomes, reducing mortality to approximately 20% and significantly increasing the rate of neurological recovery, especially when administered within the first four days of symptom onset [5]. 

HSE predominantly involves the mesial temporal lobes and orbitofrontal cortex, often bilaterally but asymmetrically. Pathologically, the infection produces neuronal necrosis, inflammatory infiltration, microhemorrhages, and cerebral edema, followed by astrocytosis and gliosis [3,6]. Histologic findings may include eosinophilic Cowdry type A intranuclear inclusions. Clinically, it presents acutely or subacutely with fever, altered mental status, seizures, focal deficits, and behavioral changes. Olfactory hallucinations may precede other neurologic signs in up to 50% of cases, although oral herpes is uncommon [3,6]. 

Cerebrospinal fluid (CSF) analysis typically shows lymphocytic pleocytosis, elevated protein, and normal glucose, sometimes with red blood cells. While viral cultures are positive in only approximately 5% of cases, polymerase chain reaction (PCR) for HSV-1 DNA in CSF remains the gold standard, with sensitivity and specificity >95% [6,7]. MRI is more sensitive than CT, revealing T2/FLAIR hyperintensities in the medial temporal lobes and limbic system. Electroencephalography (EEG) often shows diffuse slowing, although its specificity is limited [6]. 

Despite appropriate antiviral therapy and intensive supportive measures, many pediatric patients with HSE develop ICP as a result of widespread cerebral inflammation, cytotoxic edema, and hemorrhagic necrosis. Studies suggest that up to 70% of severe encephalitis cases in children may develop elevated ICP, a major contributor to morbidity and mortality [8,9]. Current guidelines for pediatric traumatic brain injury recommend a tiered approach to managing ICP, including head elevation, sedation, ventilation (target PaCO₂ 30-35 mmHg), hyperosmolar therapy (mannitol or hypertonic saline), neuromuscular blockade, and, in severe cases, barbiturate coma or therapeutic hypothermia [10]. However, in cases of refractory intracranial hypertension (rICP), where ICP remains uncontrolled despite maximal medical therapy, decompressive craniectomy (DC) has been proposed as a salvage intervention. Risk factors for developing rICP in pediatric HSE include younger age, delayed initiation of antiviral therapy, extensive bilateral or hemorrhagic involvement on neuroimaging, and the presence of status epilepticus at onset [11].

Although strong evidence for DC in HSE remains limited, several pediatric and adult case reports and small series have demonstrated favorable outcomes. A systematic review by Pérez-Bovet et al. on DC for infectious encephalitis described an 81% rate of favorable outcomes, with better prognosis in viral (primarily HSV) etiologies than in bacterial infections [12]. Similarly, Singhi et al. and Bayram et al. reported successful outcomes following DC in children and adolescents with HSE and rICP [13,14]. Multicenter pediatric experiences have shown that DC performed after day 5 of illness may be associated with survival, though neurologic deficits remain common, especially when antiviral therapy is delayed [15]. 

Following decompression, management of the autologous bone flap remains an important consideration, as it affects both short- and long-term outcomes. Various strategies exist, including cryopreservation, subcutaneous abdominal storage, and the use of synthetic substitutes, each with its own advantages and limitations. In pediatric patients, where skull growth and cosmetic outcomes are particularly relevant, identifying the most effective method for bone flap preservation is crucial. Biologically favorable bone flap preservation maintains structural viability, reduces infection risk, and enables successful later cranioplasty, particularly important in growing children. In our case, abdominal storage of the bone flap provided a cost-effective, immediately available, and well-tolerated solution that facilitated safe reintegration at three months postoperatively. 

We report the case of a five-year-old girl with HSE complicated by refractory cerebral edema and intracranial hypertension, unresponsive to maximal medical therapy, successfully treated with DC. This case adds to the growing but limited body of evidence supporting DC as a life-saving option in pediatric HSE and highlights the role of timely surgical decompression and biologically favorable bone flap preservation in selected cases. 

## Case presentation

Ethical approval 

Ethics approval was not required for this single case report in accordance with the local institutional policy. Written informed consent was obtained from the patient’s mother/legal guardian for the publication of de-identified clinical information and associated images. All efforts were made to preserve the anonymity and privacy of the patient. No identifying personal data has been included in this manuscript. 

A five-year-old girl presented with involuntary facial movements and a fixed gaze. She was born at 40.5 weeks of gestation via vaginal delivery to an adolescent mother, with no prenatal, perinatal, or postnatal complications. Birth weight was 3.23 kg. Her history included three prior hospitalizations, one for presumptive dengue at 18 months and another for an upper respiratory infection at age four, and surgical repair of an umbilical hernia in September 2015. 

Presenting illness

According to the mother, while staying at a rural campground, the patient developed fever (38-38.5 °C) and mild respiratory symptoms resembling a common cold and pharyngotonsillitis, with up to six febrile spikes per day. On day seven of illness, she had five episodes of white, non-bilious emesis, and then developed a fixed rightward gaze and involuntary movements at the right labial commissure, followed by loss of consciousness. She was initially treated at the campground clinic with intravenous phenytoin, which resulted in apparent seizure cessation. She was then transferred to the district hospital and subsequently referred to our tertiary center. On arrival, the temperature was 37.5 °C. She had labored breathing and green nasal discharge. Hydration and perfusion were adequate. Neurologically, she was disoriented but awake; her speech was clear yet incoherent. No focal motor deficits were evident. Glasgow Coma Scale (GCS) at admission was 10/15.

Laboratory evaluation revealed the following: complete blood count showed leukocytosis (12.6 × 10³/µL; reference range: 4.0-11.0 × 10³/µL) with neutrophilic predominance, hemoglobin of 11.5 g/dL (reference: 12.0-16.0 g/dL), and platelet count of 215 × 10³/µL (reference:150-450 × 10³/µL). Serum chemistry was notable for mild hyponatremia (Na⁺ 134 mmol/L; reference: 135-145 mmol/L), normal glucose (4.9 mmol/L; reference: 3.9-5.6 mmol/L), and mildly elevated hepatic transaminases, including aspartate transaminase (AST) of 42 U/L (reference: 10-40 U/L) and alanine transaminase (ALT) of 38 U/L (reference: 7-35 U/L).

Pertinent history and examination

Positive history included a product of adolescent pregnancy; term vaginal birth with normal birth weight; prior admissions for presumptive dengue and upper respiratory infection; one week of cold-like symptoms and pharyngotonsillitis treated with antibiotics; fever 38-38.5 °C with ~6 daily spikes for one week; and fixed gaze and involuntary right labial movements, followed by loss of consciousness. Positive exam findings included green nasal secretions and clear but incoherent speech. In summary, this was an acute febrile illness with neurological deterioration. 

Imaging and Initial Management

**Figure 1:**
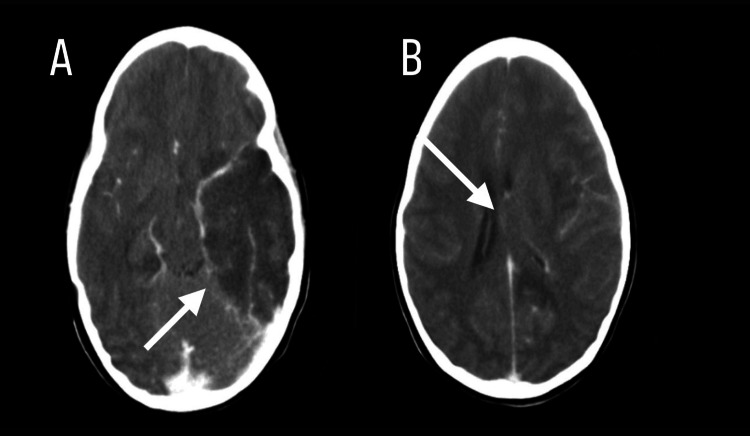
1A: CT showing extensive ischemic reaction in the left temporal lobe ( indicated by the white arrow). 1B: CT performed showing collapse of the ventricular system (indicated by the white arrow).

On the eighth day of illness, a non-contrast CT was performed, revealing an extensive hypodense, triangular lesion in the left temporoparietal region, consistent with ischemic/necrotizing injury at that level, with compression of the frontal horn, collapse of the remaining ventricular system, and obliteration of the left perimesencephalic cisterns (Figures 1A-1B). 

Provisional Diagnosis

Viral encephalitis was suspected based on antecedent respiratory symptoms, high fever, vomiting, focal neurological signs (fixed gaze, labial twitching with deviation, incoherent speech), and seizure-like activity. 

Given concern for HSE, intravenous acyclovir was initiated at 10 mg/kg every eight hours for a total of 21 days in accordance with standard guidelines for HSV encephalitis. Other potential causes, including bacterial meningitis, autoimmune encephalitis, and metabolic encephalopathies, were considered. Empiric antibiotics were initially contemplated but were deferred once clinical and laboratory findings strongly supported viral (HSV) encephalitis. To manage intracranial hypertension, sedation was started with propofol 2-5 mg/kg/h (IV infusion) and midazolam 0.05-0.2 mg/kg/h (IV infusion). Mechanical ventilation targeted PaCO₂ ~30 mmHg to induce moderate hyperventilation and reduce cerebral blood volume and ICP. Hyperosmolar therapy included 3% hypertonic saline (2-4 mL/kg bolus, then continuous infusion as needed) and mannitol 1 g/kg IV every four hours, titrated to osmolar gap and renal function. Lumbar puncture was deferred due to the high risk of herniation in the setting of mass effect due to cerebral edema and imaging evidence of elevated ICP. With clinical deterioration, an intraparenchymal ICP monitor was placed on day 8 of illness; EEG showed diffuse slowing without epileptiform discharges. Over the next 24 hours, ICP rose to 60 mmHg, necessitating barbiturate-induced coma with pentobarbital (loading 5-15 mg/kg IV, followed by 1-5 mg/kg/h infusion titrated to EEG burst suppression). Six hours later, despite EEG suppression, ICP persisted >30 mmHg. With failure of maximal medical therapy, multidisciplinary consensus recommended left-sided decompressive hemicraniectomy as a life-saving intervention. 

Surgical details and outcome

**Figure 2:**
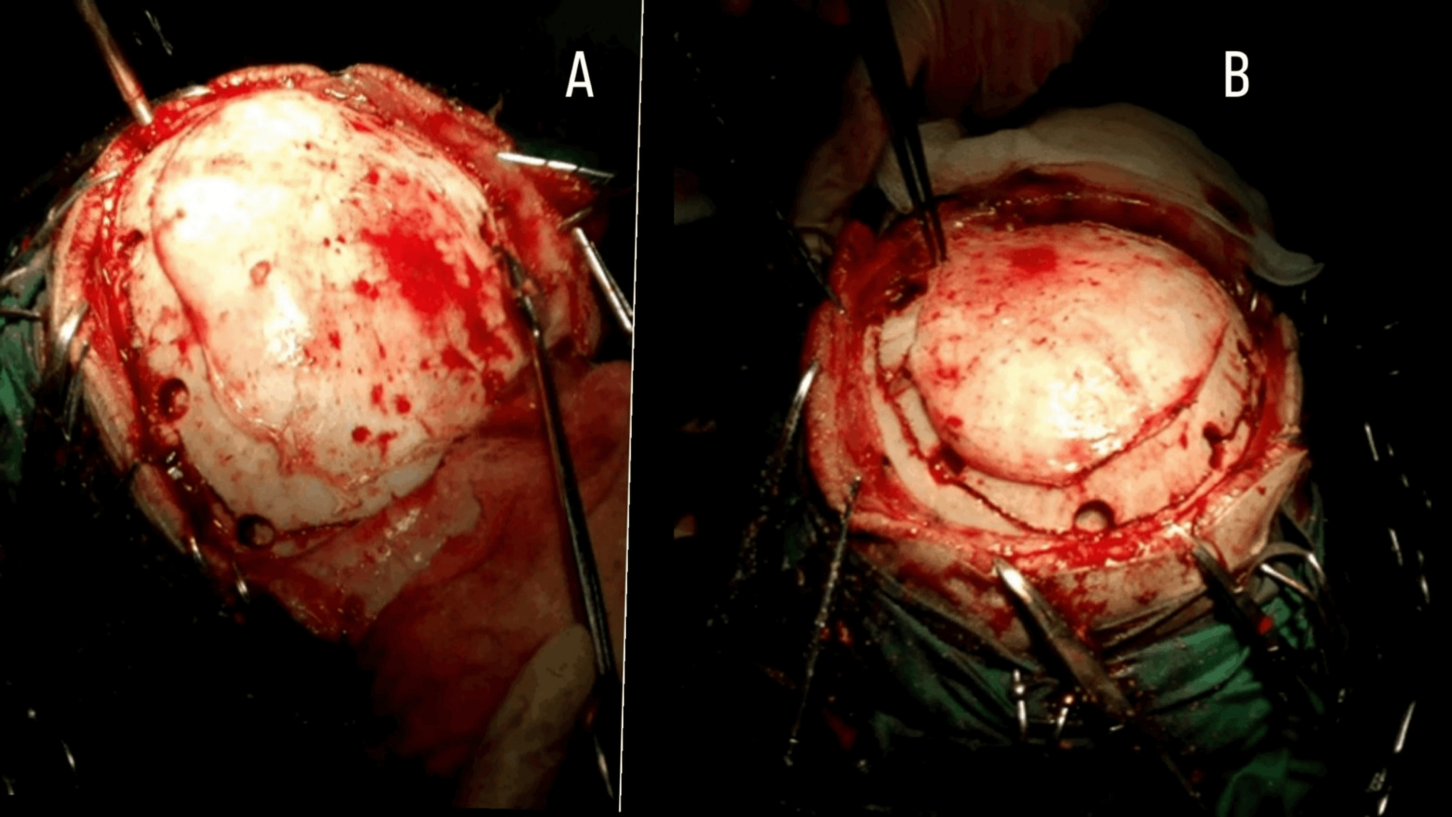
2A: Intraoperative image demostrating a careful skin incision with preservation of the temporal muscle and fascia, which are then reflected anteriorly to expose the surgical field. 2B: Subperiostal dissection revealing the anatomical landmarks used for burr hole placement:the keyhole region, the root of the zygomatic arch and the superior margin of the planned craniotomy site; the burr holes are then connected to perform the craniotomy.

**Figure 3:**
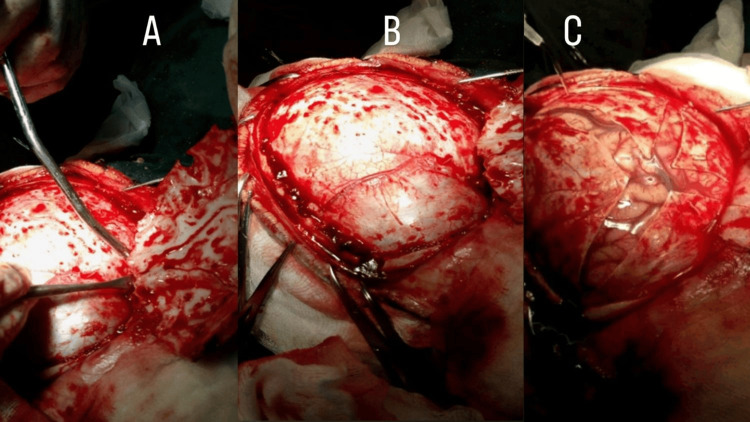
3A: Exposure of the dura mater is performed, with careful reflection of the dural flaps to expose the underlying cerebral cortex. 3B: Intraoperative view showing the brain surface after completed dural exposure. 3C: Stellate dural opening; the brain appears swollen with no significant collection requiring drainage.

**Figure 4:**
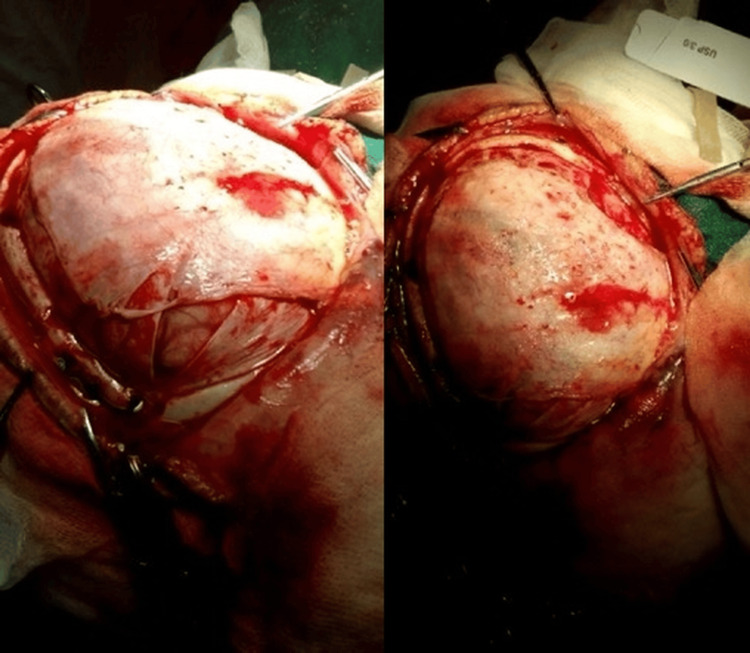
Performing a tension-free duraplasty to allow for brain expansion.

On day 9 of illness, a wide left frontotemporal-parietal craniectomy (bone flap approximately ~12 cm at greatest dimension) (Figure 2A-2B) with stellate dural opening (Figure 3A-3C) and non-restrictive duraplasty was performed (Figure 4), ensuring non-restrictive expansion to accommodate postoperative cerebral edema, enabling cerebrospinal fluid decompression and brain relaxation.

**Figure 5:**
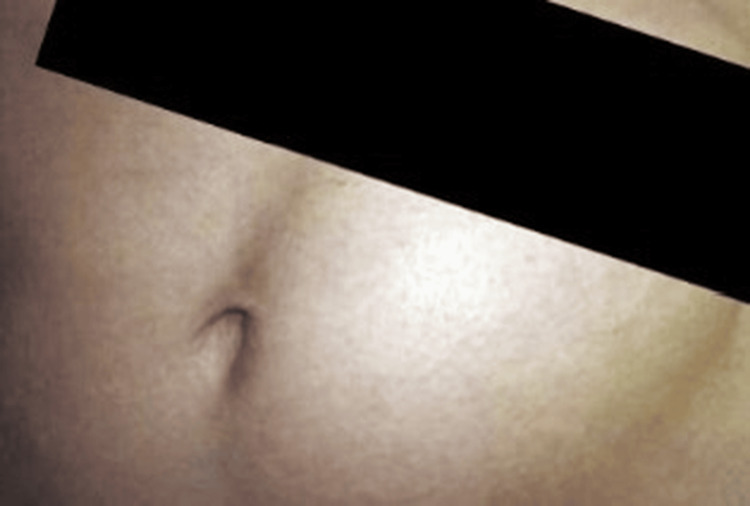
Bone plate in the left paraumbilical region.

Temporal lobe biopsy confirmed necrotizing hemorrhagic encephalitis, and PCR was positive for HSV-1. The bone flap was aseptically preserved in a left paraumbilical abdominal subcutaneous pocket (Figure 5) as a deliberate strategy to maintain autologous bone viability and structural integrity in a biologically favorable environment, reduce infection risk compared with external cryopreservation, ensure immediate availability for reconstruction, and optimize cosmetic and functional outcomes in a pediatric patient with severe, refractory ICP and impending herniation. Daily clinical evaluations and wound inspections were performed to monitor flap viability, signs of infection, or complications during the storage period.

**Figure 6:**
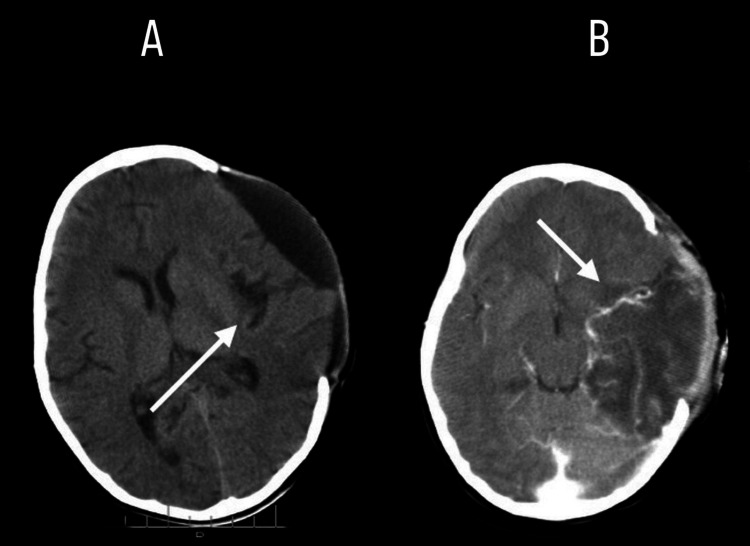
(Postoperative CT) 6A: Displayed diffuse hyperintensity on T2/FLAIR in left temporal lobe (indicated by the white arrow). 6B: Improvement of edema, and stabilized midline structures post-DC (indicated by the white arrow).

Postoperatively, ICP control was achieved with clinical and radiologic improvement. Mechanical ventilation was required for four days; ICU stay lasted seven days. A postoperative CT scan was performed, showing adequate decompression and resolution of mass effect (Figure 6).

**Figure 7:**
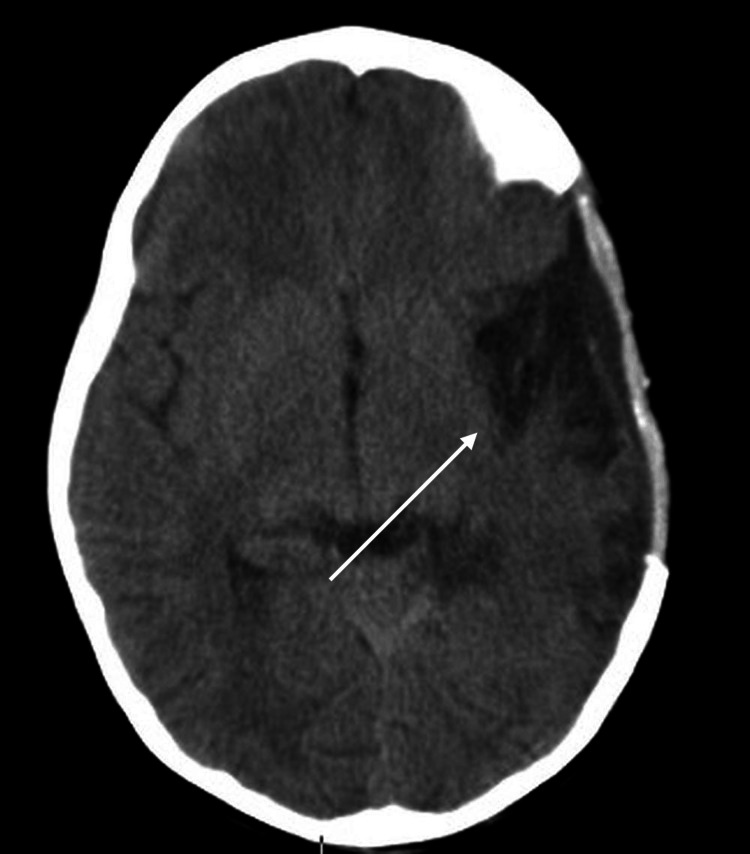
Postoperative CT at five months showed a stable craniectomy site with successful bone flap reinsertion and minimal residual encephalopathic changes (indicated by the white arrow).

At the three-month follow-up, the patient demonstrated complete physical and neurological recovery (GOS = 5, mRS = 0). Reimplantation (cranioplasty) of the autologous bone flap was successfully performed successfully at this time, allowing resolution of cerebral edema, stabilization of ICP, and reorganization of ventricular and cortical structures, minimizing risks of brain compression, flap deformation, or complications associated with delayed reconstruction (e.g., bone resorption or infection); current literature supports three months as an optimal timing for autologous bone reinsertion to balance safety and effectiveness (1-2). At five months postoperatively, a follow-up CT scan was performed, showing stable bone flap position, normal brain parenchyma, and no residual edema or mass effect. The patient continued to demonstrate full neurological recovery (Figure 7).

We believe that without decompression, the patient would likely have progressed to brain herniation and death.

The timeline of events is shown in Table 1.

**Table 1:** Timeline of events

	Event
Day 1	Fever onset and mild respiratory symptoms at a rural campground.
Day 7	Neurological deterioration: fixed gaze, involuntary facial movements, vomiting, loss of consciousness; initial treatment with IV phenytoin at the campground clinic.
Day 8	Transfer to district hospital; subsequently referred to tertiary center; a non-contrast CT was performed, showing a left temporoparietal lesion.
Day 9	ICP monitor insertion; continued sedation, hyperosmolar therapy, and ventilation.
Day 10	Left-sided decompressive hemicraniectomy performed; bone flap preserved in abdominal subcutaneous pocket; temporal lobe biopsy obtained.
Postoperative period (immediate)	ICP control achieved; mechanical ventilation for four days; ICU stay seven days; postoperative CT scan was performed, confirming adequate decompression and brain relaxation.
Month 3 (pre-cranioplasty)	CT scan performed to assess resolution of edema and plan cranioplasty; cranioplasty with autologous bone flap successfully performed.
Month 3	Neurological evaluation: GOS = 5, mRS = 0 (full recovery).
Month 5	Follow-up CT scan: stable bone flap, normal brain parenchyma, no residual edema or mass effect; patient maintained full neurological recovery.

## Discussion

HSE is the most frequent cause of sporadic viral encephalitis, and despite timely initiation of acyclovir, morbidity and mortality remain high [11]. Severe cerebral edema, hemorrhage, and necrosis may culminate in rICP, which is a critical determinant of poor outcomes. In such scenarios, no clear guidelines exist for the management of severe brain edema and increased ICP.

When maximal medical therapy, including sedation, osmotic agents, hyperventilation, and barbiturate coma, fails, DC can serve as a life-saving intervention [11,16]. Although DC is widely used in traumatic brain injury and malignant stroke, its application in infectious encephalitis is rare and supported primarily by case reports and small series [17]. Pérez-Bovet et al. reviewed 48 cases of infectious encephalitis treated with DC, reporting favorable outcomes in 81% defined as survival with functional independence, assessed by age-appropriate neurological recovery, normal cognitive and motor function, and absence of major neurological deficits at follow-up. Viral etiologies, particularly HSV, show the best prognosis [12]. Other pediatric reports, including those by Bayram et al. and Singhi et al., have also demonstrated survival and neurological recovery following DC for HSE complicated by rICP, although residual deficits remain frequent, particularly with delayed antiviral initiation or surgery [13,14]. The main published cases and outcomes are summarized in Table 2, which highlights that decompressive surgery combined with autologous bone flap preservation can result in favorable outcomes, even in severe presentations.

**Table 2:** Comparative table of published cases F: female; M: male; HSE: herpes simplex encephalitis; ICH: intracranial hypertension; TBI: traumatic brain injury

Author	Age / sex	Clinical findings	Type of DC	Bone flap storage	Outcome
Our case	5/F	HSE + herniation	Hemicranial	Abdominal	Complete recovery
Bayram 2008 [13]	11/M	Progressive HSE	Fronto-temporal	Not reported	Partial improvement
Singhi 2015 [14]	7/F	HSE + coma	Fronto-parietal	Cryopreservation	Partial recovery
Pérez-Bovet 2012 [12]	Various	Viral encephalitis	Fronto-parietal	Cryopreservation	81% good outcome
Abdul Jalal 2015 [6]	Adult/M	Progressive HSE	Decompressive	Abdominal	Good outcome
Kusulja 2018 [4]	Adult/F	HSE + coma	Fronto-temporal	Not reported	Partial recovery

Our patient illustrates these findings. Despite delayed presentation and severe rICP unresponsive to medical therapy, including hyperosmolar agents, sedation, and barbiturate coma, DC successfully controlled ICP, prevented impending uncal herniation, and allowed complete neurological recovery. This favorable evolution highlights the role of DC as a viable rescue therapy in HSE when conventional management fails [17].

A distinctive aspect of our case was the preservation of the autologous bone flap in a subcutaneous abdominal pocket. While DC is routinely performed in settings such as traumatic brain injury and malignant stroke, subcutaneous abdominal preservation of the bone flap is not routinely used in these conditions. This widely employed technique acts as a “biological incubator,” maintaining bone viability, osteogenic potential, and structural integrity, while reducing logistic and financial burdens. Compared to cryopreservation, abdominal storage may lower infection risk and facilitate reintegration, particularly in resource-limited settings. Evidence remains mixed: a systematic review and meta-analysis found no significant difference in infection rates between subcutaneous and cryopreserved flaps [18], whereas a recent randomized controlled trial reported a significantly lower infection rate with subcutaneous preservation [19], underscoring ongoing debate regarding the optimal method. The main reported benefits and evidence supporting abdominal preservation are summarized in Table 3, including its cost-effectiveness, infection safety, and biological viability.

**Table 3:** Evidence summary HSE: herpes simplex encephalitis; ICP: intracranial pressure; DC: decompressive craniectomy

Variable	Reported findings
HSE mortality without treatment	Up to 70%
Mortality with early acyclovir	Reduced to ~20%
Incidence of elevated ICP in HSE	Up to 70% in severe cases
Favorable outcome with DC	81% (Pérez-Bovet et al.)
Optimal bone flap reimplantation timing	3 months
Advantages of abdominal preservation	Cost-effective, infection-safe, biologically viable

Although synthetic alternatives such as titanium mesh, polyetheretherketone (PEEK), or polymethylmethacrylate (PMMA) offer advantages including lower rates of bone resorption and re-operation, their higher cost and limited availability make autologous preservation a practical and viable option in low-resource settings [20]. In our case, abdominal preservation allowed safe autologous cranioplasty three months later, with excellent cosmetic and functional outcomes, and without complications such as infection or resorption. The current literature supports three months as an optimal interval for reimplantation, balancing safety with favorable neurological and aesthetic results (Table 3).

This report emphasizes two key lessons. First, DC should be considered as a rescue therapy in pediatric HSE complicated by rICP refractory to medical treatment, as it may be life-saving and allow for full neurological recovery [16]. Second, bone flap preservation in an abdominal pocket remains a simple, cost-effective, and biologically favorable method for delayed cranioplasty, particularly valuable in pediatric patients and resource-constrained settings.

Finally, this report contributes to the limited literature on DC in encephalitis, particularly in the pediatric population. Few prior reports have described the use of DC for viral encephalitis with concurrent abdominal bone storage. Our case adds valuable clinical evidence supporting the feasibility, safety, and effectiveness of this combined approach.

## Conclusions

The successful neurological recovery observed in our patient highlights the life-saving potential of DC as a rescue strategy for rICP secondary to HSE when conventional therapy fails. Preservation of the autologous bone flap in a subcutaneous abdominal pocket proved to be a safe, biologically viable, and cost-effective alternative to cryopreservation for delayed cranioplasty, particularly valuable in pediatric patients and resource-limited settings where bone viability and cosmetic outcomes are critical. It should be emphasized that this report describes a single case, and its findings may have limited generalizability. Long-term follow-up demonstrated maintained neurological function and favorable cosmetic outcomes, supporting the feasibility of this approach. This case contributes to the growing evidence supporting surgical intervention in severe viral encephalitis and underscores the need for further prospective and multicenter studies to better define optimal timing, patient selection, and long-term outcomes of DC and bone flap preservation in children.
